# Autonomously shaping natural climbing plants: a bio-hybrid approach

**DOI:** 10.1098/rsos.180296

**Published:** 2018-10-24

**Authors:** Mostafa Wahby, Mary Katherine Heinrich, Daniel Nicolas Hofstadler, Ewald Neufeld, Igor Kuksin, Payam Zahadat, Thomas Schmickl, Phil Ayres, Heiko Hamann

**Affiliations:** 1 Institute of Computer Engineering, University of Lübeck, Lübeck, Germany; 2 School of Architecture, Centre for IT and Architecture, Royal Danish Academy, Copenhagen, Denmark; 3 Department of Zoology, Artificial Life Lab, Karl-Franzens University, Graz, Austria; 4 Department of Computer Science, Paderborn University, Paderborn, Germany; 5 Cybertronica UG, Stuttgart, Germany

**Keywords:** bio-hybrid, self-organization, natural plants, distributed control, adaptive construction, biotechnology

## Abstract

Plant growth is a self-organized process incorporating distributed sensing, internal communication and morphology dynamics. We develop a distributed mechatronic system that autonomously interacts with natural climbing plants, steering their behaviours to grow user-defined shapes and patterns. Investigating this bio-hybrid system paves the way towards the development of living adaptive structures and grown building components. In this new application domain, challenges include sensing, actuation and the combination of engineering methods and natural plants in the experimental set-up. By triggering behavioural responses in the plants through light spectra stimuli, we use static mechatronic nodes to grow climbing plants in a user-defined pattern at a two-dimensional plane. The experiments show successful growth over periods up to eight weeks. Results of the stimuli-guided experiments are substantially different from the control experiments. Key limitations are the number of repetitions performed and the scale of the systems tested. Recommended future research would investigate the use of similar bio-hybrids to connect construction elements and grow shapes of larger size.

## 1. Introduction

Organisms coupled with autonomous systems in an engineered bio-hybrid—that is, in closely interacting natural and technological systems—can grow into desired shapes. This could lead to alternative methods of manufacturing by shaping natural plant growth. We incorporate plants’ natural adaptive behaviours into an engineered task by expressing appropriate stimuli. From a potentially long list of possible options (e.g. mechanical, chemical and electromagnetic), we select certain spectra of light as stimuli. Appropriate sensor and control technology coupled with plants creates a system that grows user-defined shapes. Specifically, mechatronic nodes equipped with sensors and high-power LEDs operate autonomously to steer the directional growth behaviours of natural climbing plants. There is potential for a symbiotic relationship between hardware and plants, in which hardware supports plant decision-making in early growth phases, and later the matured plants mechanically support hardware. As the creation of this bio-hybrid system involves distant disciplines, the experiments are pluralistic, uniting basic protocols from plant science and engineering. Artificially extending natural growth processes requires methods that are not currently developed. Shaping plants autonomously is a fundamentally distinct task from automation of standard gardening practices (as done, for example, in the automation of greenhouses). The research presented here is an initial development towards stimuli-driven shaping of the tissues of climbing plants with mechatronic nodes, for the application of growing useful structures. Key limitations here are the scale of the natural and mechatronic systems, and the number of repetitions possible with the current experiment overhead. The approach must be extended in the future, if it is to encompass larger set-ups with more configuration flexibility and longer growth periods.

For development towards this challenging application, simple foundational methods need to be examined. Our scenario is thus simplified to hardware components readily available via current technology, but still contains the major challenges to be faced in later work towards bio-hybrid construction with plants. We grow the plants in a plane; that is, we reduce the task to two dimensions (2D) instead of three dimensions (3D) to limit the possible directions of growth. In addition, we limit the number of mechatronic nodes and the dimensions of the set-up, and place the nodes in a predefined pattern—a diagrid (figures 9*c* and 12). We use herbaceous climbing plants and grow them along the diagrid, limiting the plant’s decisions to binary left-right decisions that are uncomplicated to observe and verify. Approaches exist for automation of home gardening [1], and for autonomous systems that increase agricultural and greenhouse efficiency [2–5]. Stimuli-driven shaping technology, by contrast, must balance healthy conditions for the plant with conditions that help to direct shape. In a successful autonomous shaping system, a future user of the system would be able to input a desired shape or pattern, and the mechatronics would then ensure the correct growth. In the domain of buildings and construction, potential application scenarios depend on the time available for growing. If a long growth period (e.g. 10 years) is available, shaped bio-hybrids incorporating woody plants can potentially fulfil structural roles in buildings and infrastructure [6]. In intermediary growth periods, bio-hybrids may provide interior partition walls, or may perform standard building envelope functions (e.g. shading, wind buffering and thermal insulation). In short growth periods, they may be used as smaller fixtures, such as benches. For these applications, the degree to which a user can define or strongly influence the final shape will determine the likelihood of the technology’s adoption by architects and builders (cf. design phases management [7]). The primary application for autonomous plant shaping is the growth of building components, such as benches or green walls, that adapt to users and environmental features and can continuously self-repair. Eventual versions of this technology could be used to grow houses and even future green cities.

## 2. Related work

Autonomous bio-hybrid systems have been investigated before, with notable focus on animals. For instance, in a collective bio-hybrid system of cockroaches and robots, the robots were able to influence the social behaviour of the cockroaches [8,9], whose aggregation dynamics were significantly changed. A similar study was done showing robotic influence on crickets [10]. In larger animals, robotic interaction has been demonstrated with young chickens [11]. Forms of communication between robots and animals were further developed in the project ASSISI|_*bf*_, specifically in bio-hybrid systems of robots with honeybees [12] and robots with zebrafish [13].

Plants generally grow and move slowly, compared to the quick mobility of animals. Plants adapt their shape during growth according to surrounding environmental conditions [14]. Their slow feedback is a challenge when combined with autonomous mechatronics in a bio-hybrid system. However, as reported in previous works [15–17], robots were able to control the directional growth of plant shoots by introducing changes to their environment. In these previous works, the light stimuli were autonomously used to shape young, unsupported plants with a stem length less than 30 cm. In this paper, a mechanical and mechatronic set-up is engineered to extend this state-of-the-art to much larger plants, demonstrated with supported climbing plants of a stem length over 2 m. This engineered system contributes to open challenges that have been previously identified for bio-hybrids in autonomous construction [18,19].

Coupling natural plants and autonomous mechatronics for long periods of time triggers the necessity of monitoring and sustaining plant health. Following common practices from indoor gardening [1,3,20], many environmental variables can be regulated to ensure the well-being of plants, see §3.3.2. Existing research extends such gardening practices, for example, to grow plants in space [21]. For more typical indoor gardening, a cognitive approach uses Artificial Intelligence techniques to treat each plant individually according to its history [22]. A well-developed outdoor approach is precision farming [2,4,5], where an array of smart sensors equipped with GPS monitors many variables (e.g. soil moisture and pH levels). These readings are combined with local and satellite imagery to ensure the soil and crops receive precisely their needed resources for each location’s optimum health and fertility.

In bioinspired engineering research, a variety of plants’ climbing mechanisms are viewed as applicable to materials and actuation [23,24], including microscopic hairs augmenting the twinning mechanism of the common bean plant, used in this paper. Climbing plants that use tendrils have been studied to develop climbing robots [25] and linear actuators [26], and those using microscopic hooks on the surface of leaves have been mimicked for a dry adhesive [27]. Engineered systems incorporating biological tissues or organisms have been developed for several robotic tasks (e.g. locomotion guidance [28]), but to the authors’ knowledge have not yet been developed for climbing—although investigation into the attachment strength of certain climbing plants has progressed [29].

**Figure 1.**
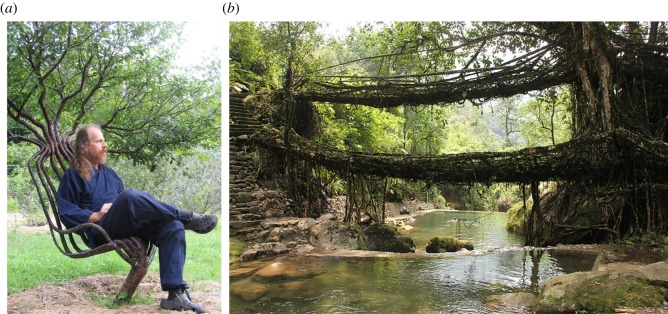
Artisan and indigenous construction of furniture and structures from living plants, using manual methods. (*a*) ‘Arborsculpture’ chair by Cook and Northey; image used with licence^2^ and (*b*) ‘Living Root Bridge’ made by indigenous construction methods in Meghalaya, India; image used with licence.^3^

Construction incorporating biological organisms has been pursued by architects and artisans (e.g. with insects, algae or fungi [30–32]). There is also existing research on self-repair of structures using biological organisms, through biocementation and bioremediation of concrete structures with certain types of bacteria [33]. These approaches have not included any autonomous technology to shape the biological material; if shaping is involved, it is enacted manually, often by moulding. Specifically, in growing structures from plants, the literature shows several artisan approaches for shaping, using combinations of mechanical constraint and rearrangement by hand (figure 1). In one approach, woody plants have been manually constrained to a building-sized mechanical frame during early growth, so that the plants’ constrained positions will form part of a façade, or grow to become part of the structural frame [34]. At the size of furniture and small products, this moulding method has been combined with grafting to achieve an agricultural process nearing mass production (see the UK firm ‘Full Grown’^1^ [35]). In another approach, woody plants have been manually constrained to one another or bundled together into small house-sized structural frames (i.e. without a separate mechanical frame), to both keep them in position and create enough stiffness for them to collectively perform a structural role [36]. This approach has sometimes been extended by natural or artificial grafting, see figure 1*a*, often termed ‘arborsculpture’ by practitioners [37–39]. Another approach, perhaps the most labour intensive, employs weekly or monthly manual rearrangement of new root growth over a time period of several decades. An indigenous technique developed in Meghalaya, India, this approach is used to construct bridges over rivers or canyons, termed ‘Living Root Bridges’ (figure 1*b*). Because of the heavy moisture and flash flooding in that area, these plant bridges have been found to outlast steel suspension bridges [6]. These various approaches give evidence for the feasibility of plants performing structural and building envelope roles. The labour-intensive processes they rely on for shaping plants could be made more convenient and scalable by introducing automation.

## 3. Methods

The autonomous system is deployed in a bio-hybrid set-up incorporating a group of natural climbing plants, a mechanical frame and a group of distributed mechatronic nodes.

### 3.1. Natural plants

Combining mechatronic and plant experiments merges standard experiment protocols from two different fields of science. The usual plant science laboratory is not designed to do robot experiments, and the usual engineering laboratory is not designed to grow plants (similarly for the experimenters). We select a plant species that grows fast enough to make the experiments feasible, and also grows reliably in an engineering laboratory. Appropriate environmental conditions (e.g. ambient light, temperature and humidity) have to be provided and then guaranteed consistently throughout the experiment. Plant growth is slow, therefore the experiments have long durations. By contrast, in mobile robotics as an example field, one is used to certain rapid-prototyping methods in experiments that even allow for a fast trial-and-error procedure when necessary. This is not available here, as the experiments are costly in overhead for an engineering task, predominantly in terms of time.

#### 3.1.1. Selection of plant species

The organisms used as biological element are climbing common beans (*Phaseolus vulgaris*). These plants grow quickly in the set-up (up to 8 cm d^−1^) and tolerate a relatively wide range of environmental parameters [40], making them straightforward plants to employ in an engineering set-up. They exhibit strong positive phototropism (i.e. directional growth towards blue light) and climb the mechanical frame set-up by twining [41]. In this experiment set-up, the beans grow in commercial soil, in 3.5 l pots (15.5 cm height, with soil level at 14.5 cm). The air temperature is kept at approximately 27°C, and the beans are watered manually on demand, as indicated by the soil moisture sensor.

#### 3.1.2. Natural climbing behaviour and circumnutation

Growing plant organs *circumnutate*—their tips move through space on helical trajectories around their mean growth direction [42]. In climbing beans and other *winders*, this behaviour is particularly pronounced, regulated and key to ecological success [41]. By rotating the orientation of its growing tip, the bean explores surroundings and winds around encountered supports. Circumnutation comes from unequal growth on different sides of an organ, which is in turn caused by reversible differences in water content of cells in the bending zone below the apex. Mechanical stimuli like gravity, pressure, shock, subsonic vibrations and tactile stimulation can influence the amplitude, speed and trace of the circumnutation [43]. When support is found, beans climb by *twining* around it in characteristic ways. The twining behaviour depends on the general environment and nutrient status, but also on the angle of inclination of the support, and on the positions and spectral qualities of incident light. If a bean does not find support after reaching a certain height, it collapses under its own weight, often in the direction of the best light. It then continues its helical upward growth from that newly supported position. In terms of plant motion behaviours, circumnutation can be classified as an autonomous active movement—it is intrinsically initiated and maintained.

Another category of plant behaviours is *tropisms*, or active directed motion and growth oriented according to environmental cues [44]. Behaviours normally operate simultaneously [45], meaning that bean shoots will at minimum balance *negative gravitropism* (growth against gravity, the most prevalent tropism of land plants [46,47]); *positive phototropism* (growth towards the light [48], explained below), and the previously described circumnutation. In other words, the shoots grow upwards, biased towards the best light, while finding and climbing surrounding supports.

#### 3.1.3. Natural behaviours for light stimuli

Employing a multitude of photoreceptors, plants can perceive light from UV-B to far-red (i.e. wavelengths 280–750 nm). Information about the abundance, direction and quality of light are tightly integrated into the development of the plant, influencing processes from germination to senescence [49–51].

In green tissues, *photosynthesis*—the light-powered production of energy-rich biomolecules from inorganic precursors of carbon dioxide and water—is driven by light from the whole visual spectrum (i.e. wavelengths 400–700 nm), with red frequencies being the most effective [52]. *Positive phototropism*, or growth towards light, is triggered by UV-A and blue light (i.e. 340–500 nm) in growing tips of many flowering plants. By emitting light in this range, the mechatronic nodes presented here attract bean shoots to climb particular rods. Cells at the surface of certain stem tissues (e.g. near growing tips or the base of leaves) are equipped with light-receptor proteins called *phototropins*, which absorb photons of energy levels corresponding to wavelengths 340–500 nm. If a tip grows towards a light source, receptors on all sides of the stem will be triggered similarly, and the growth direction will be maintained. However, if one side of the stem is significantly more exposed to the light source, water is preferentially moved to the opposite side of the stem, causing swelling. This leads to a quick and reversible bend towards the light source. As the signal persists, the flow of the major plant patterning hormone *auxin* is similarly directed. The auxin concentration fixes the asymmetry of the tissues, as they begin to stiffen and the flexible *growth zone* moves up to younger cells [48,53,54].

Plants sense whether they are being shaded, or surrounded by other plants, by the incident ratio of red to far-red light. This determines in which of two forms the cytoplasmic protein *phytochrome* is present [49]. Because plants’ green tissues absorb red light efficiently but primarily pass or reflect far-red light (i.e. near-infrared light, 700–800 nm wavelengths), a low red : far-red ratio indicates the presence of nearby plants. In some species, this cue triggers the *shade avoidance* response, where a plant tries to outgrow its neighbours via stem elongation at the expense of root and leaf formation [55]. Typically, existing leaves are also tilted upward to better compete for light exposure and dominance in a crowded environment. Shade avoidance is a complex developmental response of the plant as a whole; the term denotes an integrated response to various environmental cues with appropriate behaviours—including phototropism—to best escape shade [56]. The mechatronic nodes described here are equipped with far-red LEDs, which may augment the shaping abilities of the nodes by enabling negative feedback to the plants. This behaviour remains to be studied in future work.

Green light can be well used by plants for photosynthesis. Similar to far-red light, green light is well reflected by plants, and may also provide cues for shade avoidance [57]. To keep experimental conditions as controlled as possible for the engineering task at hand, we exclude green light, maximizing the difference in wavelength between the red light used for photosynthesis and the blue light used for phototropism.

#### 3.1.4. Natural branching behaviours

Plants may form branches from the axils of leaves, where a population of stem-cells called the axillary meristem (AM) either produces a leaf or goes into dormancy as an axillary bud. Typically, a growing tip suppresses the outgrowth of new branches. This *apical dominance* is mediated by the production and upstream transport of auxin from successfully growing tips [58]. It is also mediated by root-borne levels and downstream transport of *strigolactones*, another class of plant hormones [59]. If a dominant tip ceases its growth, or if other conditions favour it, the dormancy of axillary buds is broken. The AM then develops into a *shoot apical meristem* (SAM), the stem-cell population below a growing tip. This forms a new shoot apex, or branch. Branching is favoured under rich nutrient regimes and light with a high red : far-red ratio.

### 3.2. Distributed mechatronic nodes

**Figure 2.**
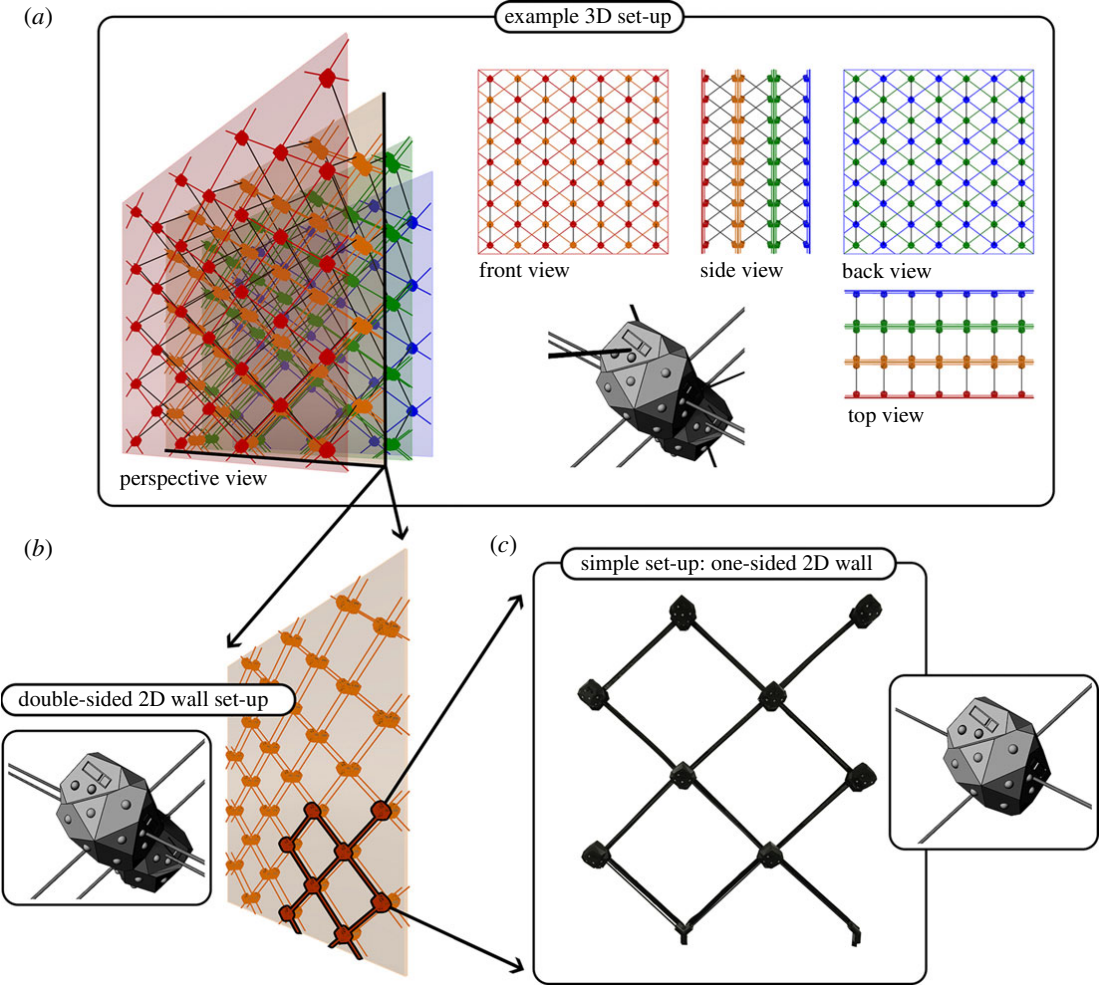
The mechatronic node is generalized to accommodate large-scale 3D set-ups, with nodes placed back-to-back in a mechanically modular diagrid frame, which can be freely reconfigured into user-defined 3D patterns. (*a*) An example set-up of back-to-back nodes integrated into a mechanical 3D diagrid. Example set-up has nodes arranged in 2D diagrids along four equidistantly spaced planes (red, orange, green and blue), with 3D diagrid connections occurring between neighbouring planes. Two of these 2D diagrids are one-sided (far-left, red; far-right, blue), the others are two-sided (centre-left, orange; centre-right, green). Because it is a 3D diagrid, in each second plane the respective 2D node positions are translated along both axes, by half the width and length of a diagrid cell. In this example, two planes have nodes in starting 2D positions (far-left, red; centre-right, green) and the other two have nodes in translated 2D positions (centre-left, orange; far-right, blue). The translated positions, a feature of a 3D diagrid, allow all rods to be of the same length (cf. rods visible in side view and those in front/back views). The experiments reported in this paper are conducted in a set-up with dimensionality reduced to 2D, and on a single-sided set-up reduced in scale. (*b*) An example set-up of back-to-back nodes in a double-sided 2D diagrid set-up, of the same scale and 2D node positions as a plane from the example 3D set-up (centre-left, orange). (*c*) The simple set-up used in the experiments herein; a small group of nodes in a single-sided 2D diagrid. This set-up matches a portion of the larger double-sided set-up (*b*, orange, highlighted in black).

As mentioned earlier, we choose a simplified set-up for these initial experiments, testing the essentials of the methodology. The mechatronic nodes, however, are designed to be general, such that they would be viable when scaling up to a larger group and adding a third dimension. Though the primary objective is to shape plants with autonomous mechatronics, the application domain also requires a structural typology that would be sensible for buildings. We follow the structural engineering literature and use the diagonal grid (diagrid) type, notable for its structural efficiency, sustainability and configuration flexibility [60–62]. Each mechatronic node has three possible diagrid attachment locations for approaching plants, and three for departing plants. Each location is equipped with the respective sensors and LEDs for its function. In more extensive set-ups, two nodes can be affixed back-to-back to receive a combination of six attachments for approaching plants, and six for departing (figure 2*b*). This back-to-back arrangement allows a flexible 3D set-up (figure 2*a*), with a mechanically modular 3D diagrid frame that can be arranged in various configurations without constraint. The set-up used for experiments in this paper is a one-sided 2D wall, comprised of eight mechatronic nodes and 12 diagrid rods (figure 2*c*). In this set-up, non-perimeter nodes receive two rods for approaching plants and two for departing, leaving plants with a binary decision about growth direction.

**Figure 3.**
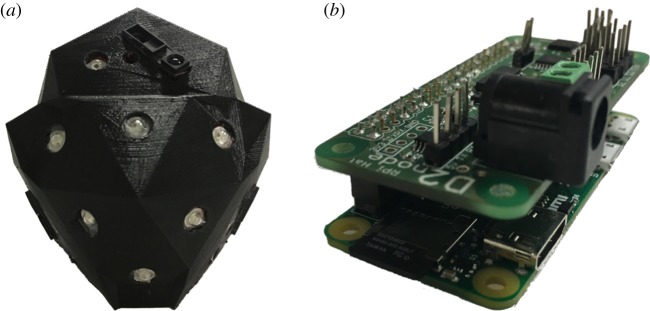
Mechatronic node for stimuli-driven autonomous shaping of climbing plants. (*a*) Node design incorporating IR-proximity sensors, photoresistors for light intensity measurements and LEDs for stimuli-driven plant actuation. (*b*) Custom Raspberry Pi HAT (i.e. PCB mounted on a Raspberry Pi Zero), interfacing with the sensors and actuators.

Each mechatronic node is approximately half-spherical in shape (≈9 cm × 10 cm × 8 cm in width, height and depth, with a flat back). It is contained in a fusion deposition modelling 3D printed polymer case faceted according to the orientation vectors of its sensors and actuators (figure 3*a*). It consists of photoresistors, IR-proximity sensors, far-red LEDs, RGB LEDs, a wireless local area network (WLAN) module and a Raspberry Pi Zero with a custom Raspberry Pi HAT—i.e. a PCB interfacing the node’s sensors and actuators to the Raspberry Pi header, shown in figure 3*b*.

#### 3.2.1. LEDs actuating plant behaviours

**Figure 4.**
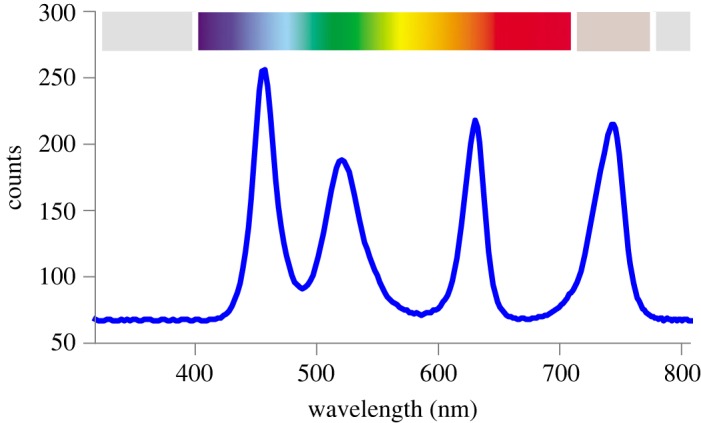
The spectrum of light able to be emitted by each mechatronic node. Peaks are blue, green, red (RGB LEDs) and far-red (λ ≈ 740 nm). The spectrum is measured at a distance of 20 cm, facing the node, with LEDs at intensity used in the experiments.

In the experiment set-up, the primary light stimulus is in the visible spectrum λ ∈ [400, 700] nm, delivered by chainable on-board Adafruit Pixie RGB LEDs. Each Pixie LED consists of a Microchip PIC microcontroller, power transistors and a high-intensity RGB LED. The RGB LEDs emit light at λ = 625, 525, 465 nm, respectively, with maximum light intensity *Φ* = 40, 55, 15 lumens, respectively. Each RGB LED has a viewing angle of 120° and a total maximal dissipation of 3 W. The range of emitted light is relevant to plants, as discussed in §3.1. The PIC microcontroller allows individual regulation of each colour’s intensity, in 32 steps. However, when set to full brightness, they quickly become hot and the PIC heat-protection mechanism turns them off until cooled. By mostly eliminating the green LED—here the least effective frequency band for phototropism, see §3.1—and by employing a DS18B20 digital temperature sensor, 3D configuration design, vents and a 5 V fan, we can achieve continuous intensities of 65% for either the red or blue LEDs. The secondary light stimulus is the far-red band λ ∈ [740, 745] nm, emitting from 3 W far-red LEDs with a viewing angle of 120° (see §3.1 for relevance to plants). Their intensities are controlled by PIC microcontrollers. Figure 4 shows the full spectrum of light emitted by a mechatronic node, as measured by a Hamamatsu C12666MA micro-spectrometer. The spectrometer values show relative intensities, labelled ‘counts’ on the vertical axis. Apart from the node stimuli, red light for photosynthesis, and a 2 s photography flash every 2 min, any other frequencies of the light in the set-up are negligible.

#### 3.2.2. Detecting approaching plant tips

Although the possible direction of an approaching climbing plant is determined, sensing its proximity is still challenging, due to the small size of the plant tip and dynamic light conditions. The experiments are visually monitored, but imaging is not used as a sensor. By using only internal sensing modules, a decentralized hardware approach is maintained. This is an important feature for self-contained products that can be replaced by non-expert users and can operate in unknown environments.

**Figure 5.**
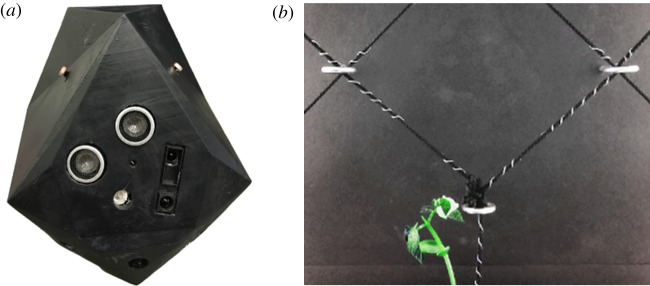
Preliminary mechatronic prototype and experiment for testing the successful IR sensing, unsuccessful ultrasonic sensing, and unsuccessful capacitive sensing. (*a*) Prototype incorporating both IR and ultrasonic proximity sensors, for comparison of accuracy. (*b*) Test of capacitive sensing, via tin wire wound onto diagrid filaments.

**Figure 6.**
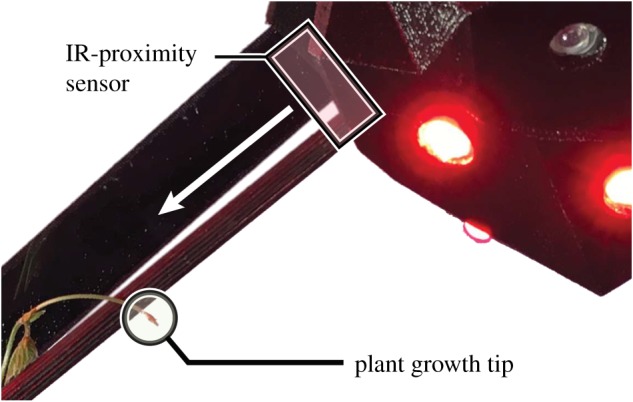
Plant climbing a diagrid rod, approaching a mechatronic node and arriving in the field of view of its IR-proximity sensor. The placement and orientation of the IR-proximity sensor in relation to the rod attachment point ensures accurate plant tip detection.

**Figure 7.**
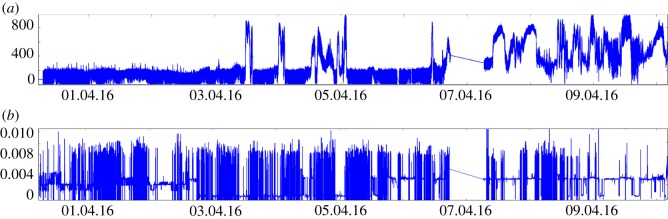
Detection data for IR (*a*) and ultrasonic (*b*) sensors in a single node. Shortly after ‘03.04.2016,’ the plant approaches the node. (*a*) Smooth and pronounced spikes are seen in the IR sensor data, when the plant tip in inside the sensor’s field of view and detection occurs. Owing to the typical winding behaviour (i.e. circumnutation) of the plant tip—which is both quick and broad—the tip moves back out of the sensor’s field of view and the IR sensor data returns to the state it held prior to ‘03.04.2016.’ The tip moves in and out of the field of view several times, with varying durations. In the IR sensor data, the difference between a present plant and an absent plant can be seen clearly. At around ‘09.04.16,’ the plant tip moves close to the sensor, which now consistently detects its presence. By comparison, no consistent pattern can be seen in the ultrasonic sensor data (*b*), as it shows no reliable reaction to the presence of a plant. This is both because the ultrasonic sensor cannot see the plant tip unless the tip provides a reflective surface oriented in a narrow angle range, and because false positives can occur from reflective surfaces on other objects.

Three different sensing methods are tested to determine the most efficient. First, capacitive sensing is tested using MPR121 sensors (figure 5*b*). In this test, filaments are mounted in a diagrid pattern for the plants to climb. Tin wire is wound onto the filaments near the diagrid intersections, and is connected to capacitive sensors. Using this set-up, the plants are correctly detected in approximately 30% of cases. Second, an early prototype of the mechatronic node, figure 5*b*, tests both HC-SR04 digital ultrasonic sensors and GP2Y0A41SK0F analogue IR-proximity sensors. The ultrasonic sensors work most successfully when facing a flat object perpendicularly, maximizing reflections. Owing to the changing orientation and small size of a plant tip, the ultrasonic sensor readings are very inconsistent. By contrast, the IR-proximity sensors achieve high accuracy. This is especially true when the direction of the approaching plant is determined (figure 6). Figure 7 gives sensor data from a plant tip approaching both an IR and ultrasonic sensor, located in a single mechatronic node. The data of the IR sensor in figure 7*a* show a clear and reliable reaction to the presence of a plant, while the data of the ultrasonic sensor in figure 7*b* show no discernible pattern. We therefore select the analogue IR-proximity sensor for the task of plant proximity detection.

In the final experiment set-up, a reading is taken every 5 s. To maximize accuracy, we develop a weighted arithmetic mean approach to detecting a plant with the IR-proximity sensor. Where the last sensor reading gives 20% of the final average, the plant tip is reliably detected at a threshold of approximately 5 cm distance from the node.

According to the sensor datasheet, the emitted IR wavelength should be restricted to 870 ± 70 nm. This is tested for confirmation. If the light spectral distribution is measured with a spectrometer less than 2 mm from the IR source, it detects some emission of critical wavelengths below 800 nm, slightly overlapping the action spectrum of the far-red LED. At distances greater than 2 mm, however, no critical wavelengths overlap this spectrum. The spectrum emitted from the IR-proximity sensor can therefore be safely assumed to not interfere with far-red LEDs that may act as stimulus.

#### 3.2.3. Communication and data logging

The current set-up uses WLAN for node-to-node communication, but emulates local communication by only allowing the mechatronic nodes to communicate with direct neighbours. The information communicated is detection of plants. The node hardware also supports communication by signal processing and photoresistors, instead of over WLAN.

The software implementation regulates uploads of sensor data and log files to a network-attached storage (NAS) device. The ZeroMQ^4^ library is used to implement communication among on-board processes and with direct neighbours. ZeroMQ is also used for logging via the network, allowing a user to visualize the stream of sensor data at run time.

#### 3.2.4. Body of mechatronic node

The mechatronic node body must allow plants to surpass it, although it serves a modest structural role as the node of diagrid [61] intersections. Because the diagrid rods are coplanar and structurally stiff, they pass through the centre of the mechatronic node. This type of connection to the node also provides some resistance to rotation when vertical loads are placed on the diagrid. Such resistance will be important in set-ups where a 3D diagrid performs structurally as a space frame. The domed, faceted shape is intended to allow the plant tip to incrementally find its way across the surface, allowing it to more easily reach frame rods on the opposite side of the node. The individual faces of the body are oriented according to the functions of sensors and actuators. Each node has four attachment points for frame rods that are coplanar (i.e. in the four corners of the node) and two attachment points for 3D frame rods projecting out of the plane, with all six rods inclined at 45° (figure 2).

**Figure 8.**
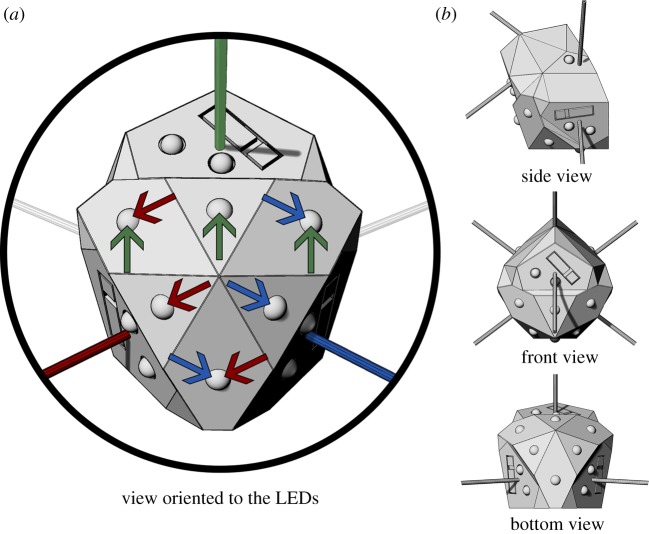
(*a*) Placement and orientation of six RGB LEDs to trigger phototropism and attract plants growing along three 3D diagrid directions. The faceted dome where the LEDs are mounted allows the surface normal of three of those faces to roughly bifurcate two of the rod directions. This is demarcated by the coloured arrows (red, green and blue) showing the rod directions serviced by each LED (e.g. LEDs with blue arrows can service the blue rod direction). This distributes the power consumption without increasing the quantity of total LEDs, and reduces the heat load on each individual LED. (*b*) Mechatronic node body from standard view planes (side, front and bottom), showing the relationship of the LED faces to the other faces of the node, and to the 3D diagrid rods.

Near the four corners of the node there are photoresistors, to detect the surrounding light conditions. Near the attachment points of each rod where plants may approach (i.e. the two lower 2D rods and the lower 3D rod) is an IR-proximity sensor for plant detection and a far-red LED for providing stimulus. Each is attached to a planar face with the surface normal aligned to the vector of the associated frame rod (figure 6). The six RGB LEDs are arranged in a triangular configuration around a faceted dome. They are positioned and oriented such that they provide a roughly equal distribution of light to the three directions of possible approaching plants (figure 8). The LED closest to each rod sits on a planar face approximately normal to the rod. Each LED sitting between two rods sits on a planar face roughly bisecting the planes normal to those two rods. In this way, the six total LEDs provide each of the three lit directions with three light sources, such that six LEDs can do the task of nine—not in intensity, but in location. Because the rods share some LEDs, the light intensity for each direction is spread over three of six LEDs rather than two of six. Assuming light intensity needed by each rod direction is constant, this positioning reduces the overall power consumption by 1/3. For instance, if each direction requires a brightness of 1.0 lumen and has two of six LEDs facing it, then each individual LED needs to provide 0.5 lumens, with the six LEDs in total needing to emit 3.0 lumens; however, if each direction has three of six LEDs facing it, then each LED instead provides 0.33 lumens, with the total needed from all six LEDs being only 2.0 lumens. Distributing the power consumption in this way helps reduce the problematic heat load on each individual LED, see §3.2.1. Limiting the quantity of LEDs also keeps the node smaller so plants are better able to bypass it.

### 3.3. Experiment set-up

In the plant binary decision wall (PBDW) set-up, the diagrid structure is arranged such that there are four potential intersections where a plant must make a binary decision—left or right—about its next growth direction.

#### 3.3.1. Plant binary decision wall

**Figure 9.**
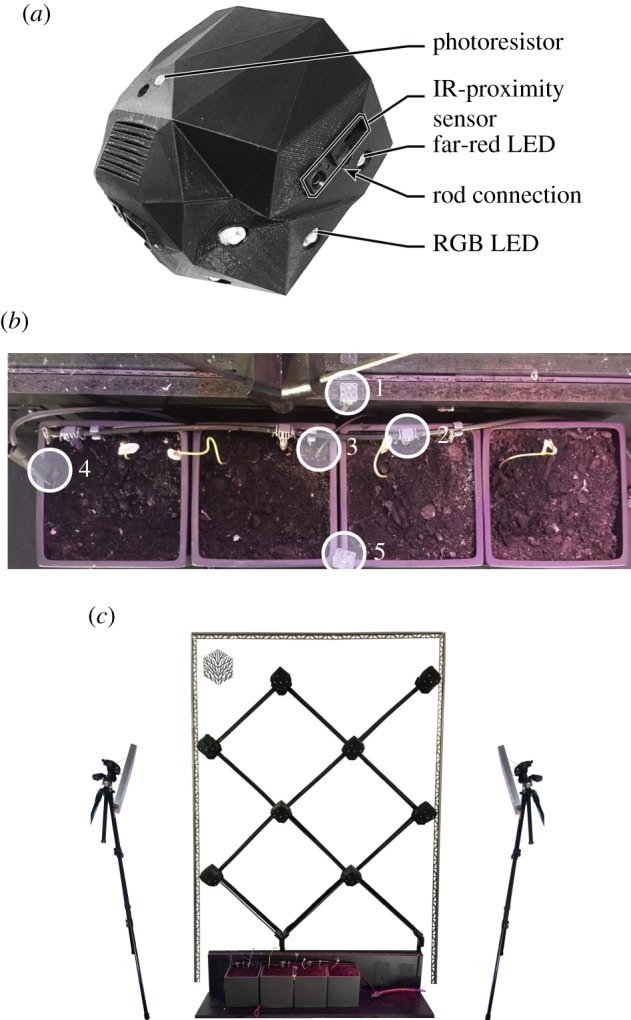
(*a*) The mechatronic node and its components, used to steer, detect, or otherwise interface with the bio-hybrid set-up. (*b*) Top view of plant shoots in their starting positions at the base of the diagrid, before an experiment is initialized, and a global environment monitor (GEM). The labelled components of the GEM are: (1) air temperature, pressure and humidity sensor; (2) water nozzle; (3) soil temperature sensor; (4) soil moisture sensor; and (5) RGB colour sensor. (*c*) The full bio-hybrid experiment set-up, before being initialized. It includes the mechatronic nodes, diagrid, plants, GEMs and supplemental growth lamps for photosynthesis.

The PBDW contains eight mechatronic nodes, see figure 9*a*, distributed in four columns and four rows, on a geometrically regular diagrid frame. The set-up includes four recently germinated plant shoots at the base of the frame (figure 9*b*). The diagrid type provides paths that are acceptable to a climbing plant, as the rods are never inclined more horizontally than 45°. For current PBDW experiments only 2D coplanar rods are used, though the node hardware and mechanics also support 3D rods (figure 2). The PBDW set-up is 125 × 0.5 × 180 cm in width, depth and height (figure 9*c*). The overall structure is climbable by the plant, meaning plants are able to bypass the mechatronic nodes that also serve a modest structural role. The mechatronic nodes are connected by 40 cm rods, inclined at 45°. The rod length is long enough to ensure that the plant is properly attached before arriving at the detection range of the mechatronic node on the other end. The coincidental detection of plant material (e.g. leaves) growing on other parts of the diagrid frame is therefore prevented. Given plant growth speeds, a plant needs an average of 8 days to fully climb one rod.

#### 3.3.2. Maintaining plant health

Plants are sensible to certain changes in their environment. For instance, while plants suffer in conditions with too little light, they are also stressed by too much light. Stressed plants slow down or even stop their growth, and eventually die. Following the biological literature [51,52,63], we build two external sensor modules—global environment monitors (GEMs)—to partially automate gardening tasks and regulate ambient light (see related work in §2). The primary GEM is operated by a Raspberry Pi 3 and equipped with a BME280 temperature-pressure-humidity sensor, a TCS34725 RGB colour sensor, a soil moisture sensor, a water pump and a camera module. It is capable of monitoring the enclosed environment where the PBDW is placed, providing the plants with water when required, and capturing close-up time-lapse videos of the experiments. Figure 9*b* shows the locations of the sensors and water nozzles. The secondary GEM is simpler; it captures time-lapse videos from a second vantage point, capturing the full wall. Similar to the mechatronic nodes, the GEMs upload the sensor data and log files to the NAS. Both GEMs run a reply server that can answer specific requests (e.g. the latest measurement of a sensor).

Two 45 W ‘Erligpowht’ LED growth lamps are added to the system to provide sufficient levels of ambient monochromatic red light, maintaining plant health during the experiments. An ‘Erligpowht’ growth lamp contains 225 LEDs—165 red and 60 blue, with peak emissions λ_max_ at wavelengths 650 nm and 465 nm respectively. Though future experiments may test ambient light conditions, here the blue LEDs on these lamps are concealed to ensure controlled laboratory conditions, preventing interference with the phototropic blue light stimulus imposed by the mechatronic nodes. At each side of the PBDW, we place one growth lamp at 90 cm height and 60^°^ inclination facing the plants, as shown in figure 9*c*.

### 3.4. Experiment types and system programming

We define two programmed states for the mechatronic nodes. In the *guiding* state, a node emits only blue light to attract the plants by triggering their phototropic response, and detects the proximity of approaching plant tips. In the *feeding* state, the node emits only red light, supporting the plants’ photosynthesis without triggering any phototropic response.

We run three types of experiments to test the performance of the bio-hybrid system. First, we run control experiments where all mechatronic nodes are set constantly to the *feeding* state, to test the growth and motion behaviour of the plants in conditions without triggered phototropism. Second, we verify the ability of the mechatronic nodes’ *guiding* state to guide plant growth to the correct target—in a binary decision between left and right—by triggering the plants’ phototropic response to blue light. Third, we test the ability of the mechatronic nodes and bio-hybrid set-up to correctly shape plant growth in a full-length experiment, into a predefined pattern on the 180 cm diagrid. All experiments include a minimum of four plants (figure 9*c*). In control, verification and predefined pattern experiments, all hardware and mechanical elements maintain uniform positions and orientations.

#### 3.4.1. Control experiments

Two control experiments are conducted with four plants each, over a total time period of about three weeks. In each experiment, the diagrid frame, nodes and GEMs are present, but all nodes are kept in *feeding* state for the duration of the experiments. The GEMs are used for monitoring and photograph documentation. Disregarding the camera flash occurring for 2 s every 2 min, there is no phototropic stimulus present in the control experiments, as both nodes and growth lamps are emitting only red light.

#### 3.4.2. Verification experiments

The verification experiments are conducted over approximately seven weeks in total, and each contains a minimum of four plants. These experiments test the ability of the distributed mechatronic system to guide the plant towards a specified target, by steering the plant’s decision-making at a given junction. This is tested in two experiments per target direction at the first diagrid junction. In each experiment, the targeted node—left or right—is set to the *guiding* state, and the opposing node is set to the *feeding* state. An experiment is considered successful if all attached plant tips find the correct rod, and only the *guiding* node detects an approaching plant. Another feature considered favourable is a high proportion of unsupported shoots growing with bias to the *guiding* node.


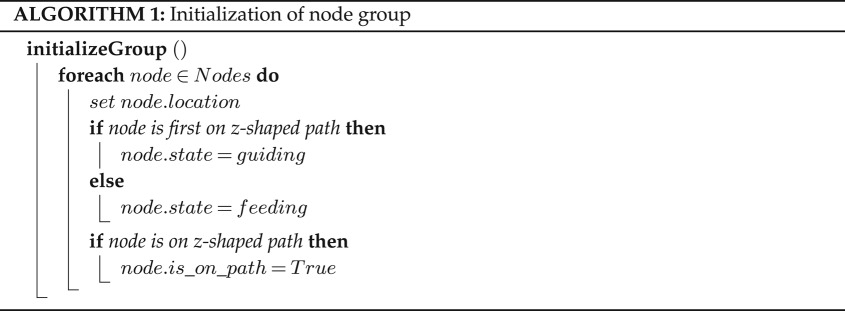



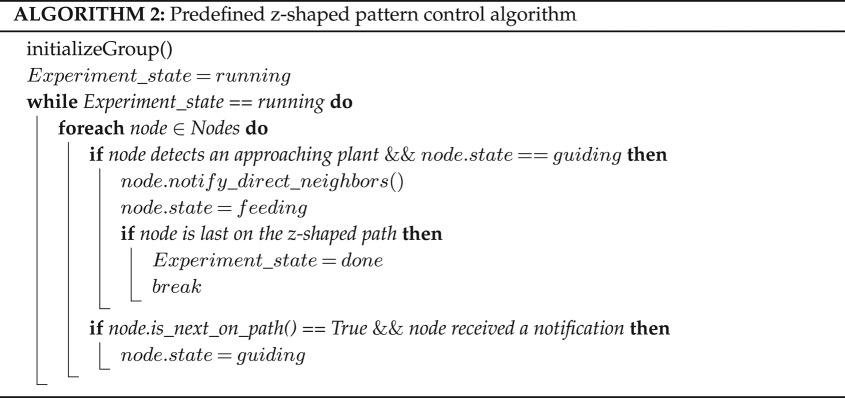


#### 3.4.3. Predefined-pattern experiments

These full-length experiments investigate the ability of the decentralized control and distributed mechatronics to grow a plant into a predefined shape. Two such experiments are conducted, over a total time period of approximately 14 weeks. In these experiments, the mechatronic nodes are programmed to grow a single plant into a given shape. However, because plant experiments are costly in time, we include four plants and collect additional information on their overall growth (see §4). In each predefined-pattern experiment, the first plant to attach to the diagrid at any point is considered the *leading* plant. The experiments then test the interaction of this *leading* plant with the mechatronic nodes, which are programmed to shape it into the user-defined pattern. The experiment ends when the *leading* plant reaches the final node in the pattern. It is considered successful if the *leading* plant consecutively reaches every node and rod in the pattern, and never attaches to an incorrect rod.

The system initializes (see algorithm 1) by providing each node with its location on the diagrid map and with a user-defined pattern (figure 12*a*). The nodes use this predefined pattern and local communication (i.e. with only their direct neighbours) to trigger their state change. Only one node is allowed to be in the *guiding* state at any given time, beginning with one on the first level. Once any node detects an approaching plant (see algorithm 2), it notifies its direct neighbours and switches its state to *feeding*. According to the nodes’ locations in relation to the pattern map, one of the nodes on the next level will then switch its state to *guiding*. This process is repeated until a node on the final level detects an approaching plant. Both of the predefined-pattern experiments are continuations of the left *guiding* verification experiments.

## 4. Results

### 4.1. Control experiments

**Figure 10.**
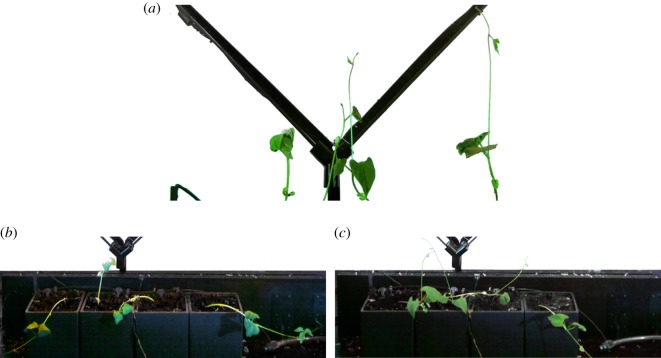
Frames of results from control experiments, where the plants are exposed only to *feeding* light spectra and not to *guiding* light stimulus. Without any *guiding* light, none of the eight plants were able to attach to a diagrid rod. (*a*) Control experiment #2. All four plants showing straight upward growth with no attachments, after 5 days. (*b*) Control experiment #1. Three of four plants collapsed, one growing upward without attachment, after 15 days. (*c*) Control experiment #2. Two of four plants collapsed, two growing upward without attachment, after 7 days.

The plants show unbiased upwards growth due to the lack of a directional phototropic stimulus by blue light, in addition to their standard gravitropism (i.e. growth against gravity), figure 10*a*. The typical circumnutation (i.e. winding motion) is observed. This autonomous behaviour is expected, as the plant explores the environment and searches for climbing support, see §3.1. However, as expected, the plants are not able to find and attach to the diagrid rods without external steering. The experiments are stopped when at least two of the four plants collapse from unsupported self-weight^5^ (figure 10*b*,*c*). None of the plants attach to the scaffold.

### 4.2. Verification experiments

**Figure 11.**
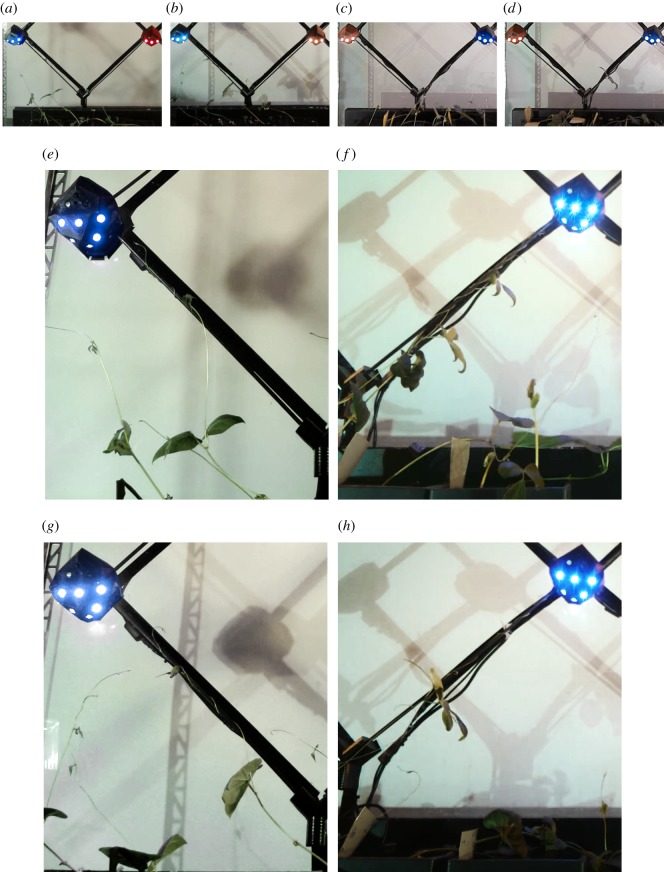
Frames of results from the verification experiments, which test the ability of the mechatronic nodes’ *guiding* state to reliably steer the plant through a binary decision, to reach the correct rod and target. In the left *guiding* experiments—#1 (*a*,*e*) and #2 (*b*,*g*)—the node left of the junction is set to the *guiding* state, with the opposing set to *feeding* (*a*,*b*). In the right *guiding* experiments—#1 (*c*,*f*) and #2 (*d*,*h*)—the node right of the junction is set to the *guiding* state, with the opposing set to *feeding* (*c*,*d*). Each frame shows the condition of growth just before the *guiding* node detects the approaching plant. In all four experiments (see *e*,*f*,*g*,*h*), at least one plant attaches to the correct rod (i.e. the one leading to the *guiding* node). None of the plants attaches to the incorrect rod. In further evidence of the nodes’ effects, the unsupported plants also generally display growth biased towards the *guiding* node. (*a*) Left *guiding* #1. (*b*) Left *guiding* #2. (*c*) Right *guiding* #1. (*d*) Right *guiding* #2. (*e*) Left *guiding* #1, close-up of figure (*a*); one week of growth; one plant attached. (*f*) Right *guiding* #1, close-up of figure (*c*); two weeks of growth; two plants attached. (*g*) Left *guiding* #2, close-up of figure (*b*); one week of growth; one plant attached. (*h*) Right *guiding* #2, close-up of figure (*d*); three weeks of growth; one plant attached.

There are two verification experiments per target (i.e. the node set to *guiding*), all occurring at the first diagrid junction. They each run continuously, for 13 days on average. In each of the four experiments, a plant successfully chooses and attaches to the correct rod, climbing it until reaching the target (figure 11). In over 90% of the unsupported shoots, the typical circumnutation motion of winding is pronouncedly biased to the target (see footnote 5). This results in consistent tilting of the upright stems towards the target, in areas where tissues have stiffened. In each experiment, the plant with stem angle and location most similar to that of the correct rod (i.e. the rod connected to the *guiding* node) is the first to attach. In each case, that first *leading* plant continues to climb the rod until it reaches the target. In one experiment, a second plant also attaches to the correct rod. The experiments are stopped once a plant reaches the target. Out of over 20 total plants in all verification experiments, none of the plants attaches to the incorrect rod (i.e. the rod leading to the *feeding* node). Taken together, these results suggest that the mechatronic nodes are capable of reliably steering the plant through a binary decision, until it reaches the specified target.

**Figure 12.**
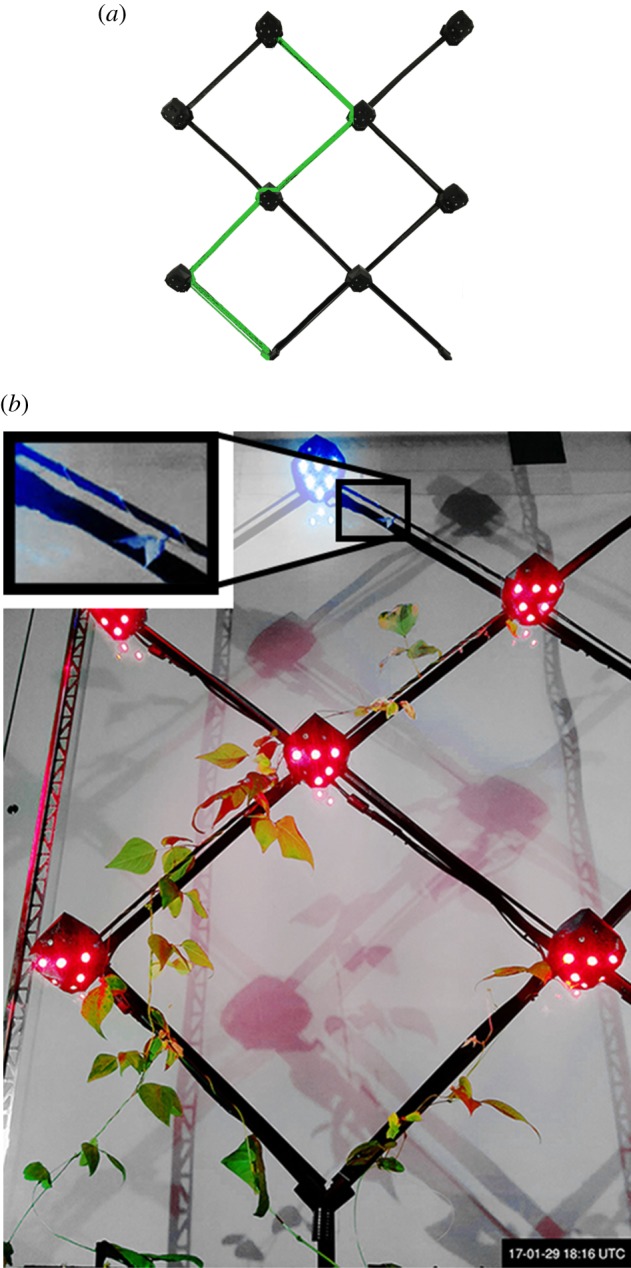
Main result; predefined-pattern experiment #2. (*a*) Visualization of the z-shaped pattern on the diagrid map. The mechatronic nodes are programmed to grow a single plant into this user-defined pattern, on the 180 cm set-up. (*b*) Frame of final experiment configuration after 40 days. In this experiment, the *leading* plant—the one which the nodes are programmed to shape—successfully fulfils the user-defined pattern. The *leading* plant consecutively grows along each diagrid rod in the pattern (towards the respective *guiding* node), and successfully triggers each node in the pattern, reaching the final *guiding* node at the top of the diagrid. The *leading* plant never attaches to an incorrect rod. The non-*leading* plants (i.e. those that climb the diagrid at a later stage in the experiment) also provide relevant results, though they are not the main result being tested in this experiment. There is a non-*leading* plant outside the pattern in (*a*) because it grows towards the concurrently triggered node. The non-*leading* plant in the lower right corner attaches to that rod because at the time it represents the most direct route to the concurrently triggered node.

### 4.3. Predefined-pattern experiments

These two experiments grow the same user-defined pattern, each running seven weeks continuously. The first experiment undergoes a minor technical glitch in the set-up, so we end it early and progress with the second experiment. The second experiment runs without issue and completes the full task. Excluding the roughly 11 h period of the technical glitch, all experimental results are consistent.

**Figure 13.**
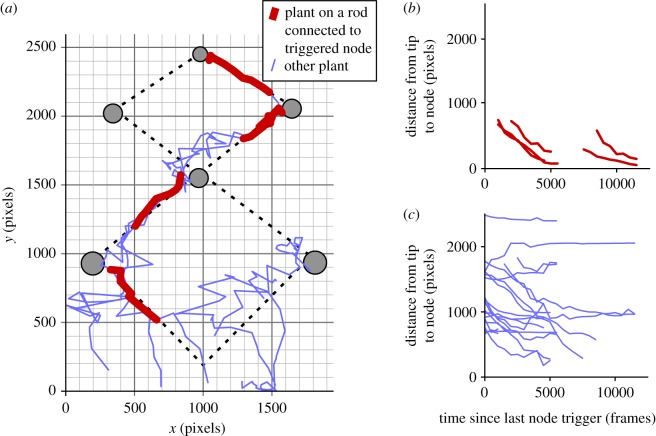
Movement of plant tips shown in experiment photographs, in the predefined-pattern experiment #2 using the Z-shaped pattern in figure 12*a*. Tip locations are observed and recorded, at 1000 min time intervals (i.e. at every 500th frame). (*a*) The *xy* movements of visible plant tips in the image frame, coloured according to whether the plant has attached to a diagrid rod that is connected to the currently triggered node (i.e. *guiding* node). (*b*,*c*) The changes in plant tips’ distance from the currently triggered node, throughout the experiment, relative to the time since the last node triggering event. For plants attached to a rod connected to the currently triggered node (*b*), a plant tip on average moves closer to the triggered node by approximately 13.0 pixels (approx. 6.4 mm) per 100 frames (i.e. 200 min). The start point of each curve in (*b*) occurs at the frame when that plant tip attaches to the relevant rod. Horizontally, one group of curves begins around the 1000th–2500th frame; this group of plant tips attaches to a new rod quickly after the last node triggering event. The other group of curves begins around the 7500th–9000th frame; this group attaches to a new rod more slowly, and is comprised of plant tips climbing around a mechatronic node to reach the opposing rod. Vertically, the start points of curves occur according to the distance of the plant tip from the triggered node when the tip attaches to the new rod. Regardless of the time and location of the tip attachment, each curve in (*b*) declines steadily, as all attached plant tips progress reliably towards the currently triggered node. For other plants (*c*), a tip on average moves approximately 5.6 pixels (approx. 2.8 mm) closer to the triggered node per 100 frames (i.e. 200 min).

In both experiments, each of the seven times a plant tip grows along a diagrid rod and approaches a node, the node successfully detects the tip using the IR-proximity sensor and the weighted mean approach, see §3.2.2. Similar plant behaviour occurs in both experiments. Here we begin by describing plant behaviour in the unimpaired experiment (i.e. predefined-pattern experiment #2, see footnote 5). Early on, the *leading* plant successfully attaches to the first correct diagrid rod and climbs it (figure 13*a*). The first *guiding* node (lower left) detects the *leading* plant when it is within range, and triggers the node on the next level. Two additional plants attach to the first correct rod and continue climbing towards the *guiding* node. By contrast, the plant located at the far right (closest to an incorrect rod) does not find any rod and continues unsupported upward growth until it collapses. The *leading* plant—followed by the two other attached plants—continues to climb the diagrid and trigger the next *guiding* node. Now, the plants have two choices: either climb along the nearby rod leading away from the target, or climb fully around the mechatronic node to bypass it and reach the correct rod. The *leading* plant takes several trials bypassing the mechatronic node. Its dominant tip then moves out of visibility, and it grows a new nearby tip, or branch, see §3.1.4, which easily bypasses the node. That is, it grows around the node with a wide exploratory margin, finding—but rejecting—the diagrid rod pointing in the wrong direction, and finally reaching—and attaching to—the correct rod on the opposite side of the mechatronic node. The two other plants easily follow the *leading* plant towards the *guiding* node on the third level. Meanwhile, the plant that collapsed earlier also grows a new tip. This non-*leading* plant attaches to a diagrid rod that is outside the predefined pattern, choosing the shortest possible path to the concurrent *guiding* node (figure 12*b*, lower right). The *leading* plant tip continues until it arrives at the final *guiding* node on the fourth level, see insert in figure 12*b*. The user-defined pattern is successfully grown via the *leading* plant (figure 12*b*), and the experiment stops. The total experiment time is seven weeks.

The results plots in figure 13 show the progression of plant tip movements during experiment #2. The *leading* plant tip successfully attaches to each of the four correct rods (i.e. those connected to the concurrent *guiding* node). The quickest attachment event is near to that rod’s beginning, and the slowest is near the middle of that rod. Other than the *leading* plant, two other plants also manage to attach to a correct rod. In figure 13*a*, we see that the plant tips take a much shorter path to the next attachment when the target rod is on the nearby side of the node. When the plant has to bypass the node to find the target rod, it searches in a larger area. Even though it passes by an easily reached rod—and the *twining* behaviour is a strong influence, see §3.1—the plant chooses to continue following the phototropic stimulus until it reaches the target rod, even if this requires a *branching* event, see §3.1.4. In figure 13*b*, we see that when plants are attached to a rod connected to the *guiding* node, the tips approach (by growth and motion) the node consistently and quickly, at an average speed of ≈ 1.9 mm h^−1^. The stimulus provided by the nodes therefore successfully completes the intended task. In figure 13*c*, we see that the attractive stimulus additionally influences other plants favourably, as even without helpful attachment they on average approach (by growth and motion) the *guiding* node at ≈0.8 mm h^−1^.

In predefined-pattern experiment #1, results similar to experiment #2 are observed, until the *leading* plant triggers a *guiding* node on the third level. Then due to a technical malfunction, an extremely intense light source normally used as a photography flash—500 W, much brighter than normal indoor ambient light—is left continuously turned on overnight. Consequently, the plant’s phototropic behaviour causes the expected growth pattern to be altered for roughly an 11 h period, as the *leading* plant grows away from the *guiding* node.^6^ After the technical issues are resolved, another plant grows towards the *guiding* node and becomes the new *leading* plant. It attempts to bypass the mechatronic node with several trials. Similar to what is observed in the other experiment, rather than choosing to climb along the closer—but incorrect—rod, the plant instead grows a new nearby tip which easily succeeds to bypass the node and reach the correct rod. The plant then triggers a *guiding* node on the fourth level, but we stop the experiment after a total time period of seven weeks due to the set-up malfunction.

In summary, we find a significant difference between the results of these experiments with a predefined pattern, and the control experiments in which no plant attachment occurs. The mechatronic system is therefore effective in shaping a plant.

## 5. Discussion and future work

### 5.1. Discussion

The results show a significant difference between the control experiments and the plant-shaping experiments. The diagrid rods were positioned some distance away from plant roots (i.e. not directly above) and were not inclined at an angle at which climbing plants naturally grow without the influence of directional (i.e. tropic) stimuli. Therefore, in the control experiments, the lack of directional stimuli caused any attachment to be impossible for the plants.

The root positions relative to the diagrid set-up had an important impact on which individual plant was the first to successfully attach. Plants positioned closer to the start point of the predefined pattern (i.e. the first correct rod) and, relative to that start point, positioned opposite the first attractive stimulus, were more successful at attaching first. A plant rooted this way grew freely for some height and found the first rod while growing towards the attractive stimulus. Its angle of inclination when its growth was unsupported and steered was similar to the rod’s angle of inclination. The plant tip therefore maintained short distances from its target in the unsupported phase. After attachment, the nodes were effective in directing the plant’s decision-making (e.g. left-versus-right decisions). The user-defined pattern was grown in the main experiment (i.e. predefined-pattern experiment #2), as seen when comparing the pattern in figure 12*a* to the results in figures 12*b* and 13*a*. Additionally, any triggered *guiding* node consistently and quickly attracted the plants attached to its connected rod, as seen in figure 13*b*.

In this experiment set-up, the *leading* plant was the main focus and was solely assessed when looking for completion of the predefined pattern. Partially, we focused on the *leading* plant in this set-up due to the complexity present in plant behaviour, as described in §3.1. When the *leading* plant triggered a new *guiding* node, the stimulus also attracted the other, non-*leading* plants. This sometimes triggered a branching event or directed extraneous attachment to diagrid rods. For those non-*leading* plants, the rods they chose were pointed most directly towards the concurrent attractive stimulus, even if they were not part of the *leading* plant’s targeted pattern. This attraction is seen in figure 13*c*, as tips that were not attached to a rod connected to the *guiding* node still on average approached that node at a rate of ≈ 0.8 mm h^−1^. One example for such possibility is observed in experiment #2, figure 12*b*, where an extraneous lower rod was populated by a plant. In the current set-up, this extraneous behaviour of non-*leading* plants is not prevented by any stimulus. It could be prevented, for example, by shading the lower parts at later stages of the experiment or by providing other autonomous repelling stimuli, such as far-red light or vibration motors [18,19]. Our favoured option currently would be far-red light, see §3.1.3, which could trigger the plant’s *shade avoidance* response. The mechatronic nodes reported here are equipped with far-red LEDs, see §3.2.1. In the current scenario—which shapes a single *leading* plant—by using correct signalling to complement the attraction stimulus, the far-red LEDs may potentially trigger a plant to not branch or attach at extraneous locations. In more complicated scenarios, other control approaches would be required—specifically, a sophisticated decentralized algorithm exploiting both shade avoidance and phototropism—in order to individually and simultaneously steer multiple plants into user-defined shapes.

In the diagrid frame used in these experiments, there are four potential locations where climbing plants make a binary decision. If a plant chooses to maintain its growth direction rather than switch, then it is required to fully climb around the body of the node in order to reach the diagrid rod on the opposite side. This is challenging, as the node could block the incoming attractive stimulus. In order to succeed, the climbing plant needs to grow some length and ignore an opportunity to attach to an incorrect rod (i.e. one leading away from the attractive stimulus), then find and attach to the correct rod behind the node. In the plant-shaping experiments reported here, this case exists between the second and third levels on the path of the predefined pattern. As discussed in §4.3, we witness this difficult task in both full shaping experiments. First, each *leading* plant took several trials climbing around the mechatronic node, each time succeeding in ignoring the incorrect rod. Subsequently, each grew a new dominant tip that—in its first trial—bypassed the node and attached to the correct rod. This process required approximately 11 days in both experiments. In comparison to normal growth speeds, this is an indication of the task difficulty, as plants were normally able to fully climb a rod in 8 days on average. This repeated rejection of an incorrect rod throughout a significantly difficult climbing task is evidence for the strength of the attractive stimulus emitted by the nodes, even after branching and in plants’ later growth stages. Interestingly, once each *leading* plant climbs around the node successfully, it creates a natural scaffold, allowing the other plants to easily climb and follow. A different mechatronic node design of smaller size, or of different incorporation into the mechanical frame, may allow the plants to climb more smoothly around it.

One important limitation here is the scale of the bio-hybrid system. Further investigation would be required to confirm the extension of the approach to large groups of plants and distributed mechatronics, in which many simultaneous plant decisions must be supported and steered. In such large systems, plant health must also be maintained for longer periods and in further developmental stages. Another important limitation is the number of repetitions performed, restricted by the high degree of experiment overhead. Both large-scale systems and increased repetitions would need to be supported by a mass production approach to the mechatronics, and by substantial greenhouse infrastructure within plant science or other biology laboratories.

### 5.2. Future work

The approach can be usefully extended by incorporating other stimuli in the control process in addition to blue light. Far-red LEDs can be investigated as repellents for plants, as combining attractive blue light and repellent far-red light could allow more nuanced control of shaping, as described above. In addition, other types of sensors (e.g. sensing sap flow or activity of photosynthesis) can be investigated, for possible addition to the mechatronic nodes. Further useful modifications would be to the node design, in order to build smaller, light-weight versions. Their mechanical integration could be extended to different types of structures, including flexible scaffolds and bigger plants. A more flexible structural scaffold could potentially be integrated with the nodes in such a way that the elements for climbing wrap along the outer surface of the node instead of passing through the centre, making it easier for a plant to climb past the node. In the work presented here, the plants do not serve a structural role, as they are fully supported by the diagrid frame. Climbing species that become woody over time can be investigated, to be shaped before the onset of lignification—before which the stem is still flexible—and then stiffened as they become woody. Such species would allow a changing relationship between mechanical elements and plants, where the mechanical serves as supporting structure in early growth stages and the plants serve as structure in later stages, creating bio-hybrids that can construct building components. An important future work can be to progress from 2D to 3D shaped plant growth, as highlighted by the node design described here.

More complex shapes and user-defined patterns might be generated by applying several simultaneous stimuli sources, and by using denser mechanical frames with more elements for climbing, extending to a gradient of choices for plant tips rather than binary decisions. Extensions to control can also allow the mechatronic nodes to adapt to measured environmental conditions. For example, the intensity of the light emitted by every node can be adapted to local ambient light. The objective is to shape the plant regardless of variations in the light conditions. Fully decentralized algorithms that run on the mechatronic nodes would allow the system to use programmed swarm intelligence to adapt to manual rearrangements of mechatronic nodes. The user could then change the topology of the node network by adding and removing them. The photoresistors can be used for local 1-bit communication to provide minimal spatial information.

With future increases in complexity, nuance of control, and scale, growth patterns could be shaped to infill a frame in desired areas, forming a façade with windows and doors, and performing building envelope roles such as wind buffering. By moving to species that become woody over time, such living constructions may also perform mechanically as benches or partition walls, eventually even forming more advanced structures for houses or bridges.

## 6. Conclusion

We have presented bio-hybrid experiments of a novel approach using autonomous distributed mechatronics to shape the growth of natural climbing plants. The mechatronics and plants closely interact. The mechatronics are effective at shaping the plants by light stimuli in properly configured indoor conditions. The mechatronics also operate autonomously based on correctly sensing the position of the plant tip. Several novel challenges in plant–robot bio-hybrid systems have therefore been addressed, including sensing plants, generating appropriate stimuli, combining mechatronic experiments with plant experiments, and integrating the distributed mechatronics into mechanical climbing support. In two pattern experiments, we successfully show the feasibility of the shaping approach, supported by further verification experiments of binary plant decisions. The two control experiments with non-reactive mechatronics and constant light conditions show a significantly different plant behaviour than the shaping and verification experiments. Key limitations are the number of experiment repetitions and the scale tested. The developed technology and results are foundational developments towards growing building components from autonomously controlled plants.

## Supplementary Material

Plant shaping experiments: Control, single-decision, and full pattern

## Footnotes

1 See https://fullgrown.co.uk.

2 Image owned by Peter Cook and Becky Northey of the firm Pooktre, retrieved from Wikimedia Commons, from username Blackash at English Wikipedia. Used with Creative Commons licence CC BY 3.0. Image copyright holders approved the licence under OTRS ticket no. 2011020310002057.

3 Image retrieved from Wikimedia Commons, attributed to Arshiya Urveeja Bose. Used with Creative Commons licence CC BY 2.0. Image copyright holder chose and approved the licence at upload.

4 ZeroMQ asynchronous messaging library http://zeromq.org/.

5 Video accessible: https://vimeo.com/246114841.

6 Video accessible: https://vimeo.com/246114841.

## Data accessibility

Experiment data are publicly accessible at Zenodo^7^: https://doi.org/10.5281/zenodo.1172160 [64].

## References

[1] Correll N *et al.* 2009 Building a distributed robot garden. In 2009 IEEE/RSJ Int. Conf. on Intelligent Robots and Systems (IROS 2009), St Louis, MO, 10–15 October, pp. 1509–1516. IEEE (10.1109/IROS.2009.5354261)

[2] Åstrand B, Baerveldt AJ 2002 An agricultural mobile robot with vision-based perception for mechanical weed control. Auton. Robots 13, 21–35. (10.1023/A:1015674004201)

[3] van Henten EJ, Hemming J, van Tuijl BAJ, Kornet JG, Meuleman J, Bontsema J, van Os EA 2002 An autonomous robot for harvesting cucumbers in greenhouses. Auton. Robots 13, 241–258. (10.1023/A:1020568125418)

[4] Blackmore BS 2007 A systems view of agricultural robots. In Proc. 6th European Conf. on Precision Agriculture (ECPA), Skiathos, Greece, 3–6 June, pp. 23–31. Wageningen, The Netherlands: Wageningen Academic Publishers (10.3920/978-90-8686-603-8)

[5] Edan Y, Han S, Kondo N 2009 Automation in agriculture. In *Springer handbook of automation*, pp. 1095–1128. Dordrecht, The Netherlands: Springer (10.1007/978-3-540-78831-7_63)

[6] Shankar S 2015 Living root bridges: state of knowledge, fundamental research and future application. In *IABSE Symposium Report*, vol. 105, pp. 1–8. International Association for Bridge and Structural Engineering (10.2749/222137815818359474)

[7] Knotten V, Svalestuen F, Hansen GK, Lædre O 2015 Design management in the building process: a review of current literature. Procedia Econ. Financ. 21, 120–127. (10.1016/S2212-5671(15)00158-6)

[8] Halloy J *et al.* 2007 Social integration of robots into groups of cockroaches to control self-organized choices. Science 318, 1155–1158. (10.1126/science.1144259) 18006751

[9] Caprari G, Colot A, Siegwart R, Halloy J, Deneubourg J 2005 Animal and robot mixed societies: building cooperation between microrobots and cockroaches. IEEE Robot. Autom. Mag. 12, 58–65. (10.1109/MRA.2005.1458325)

[10] Guerra R, Aonuma H, Hosoda K, Asada M 2010 Behavior change of crickets in a robot-mixed society. J. Robot. Mech. 22, 526–531. (doi:10.20965/jrm.2010.p0526)

[11] Gribovskiy A, Halloy J, Deneubourg JL, Bleuler H, Mondada F 2010 Towards mixed societies of chickens and robots. In 2010 IEEE/RSJ Int. Conf. on Intelligent Robots and Systems (IROS), Taipei, Taiwan, 18–22 October, pp. 4722–4728. IEEE (10.1109/IROS.2010.5649542)

[12] Mariano P, Salem Z, Mills R, Zahadat P, Correia L, Schmickl T 2017 Design choices for adapting bio-hybrid systems with evolutionary computation. In Proc. Genetic and Evolutionary Computation Conf. Companion, GECCO ’17, Berlin, Germany, 15–19 July, pp. 211–212. New York, NY: ACM.

[13] Bonnet F, Cazenille L, Gribovskiy A, Halloy J, Mondada F 2017 Multi-robot control and tracking framework for bio-hybrid systems with closed-loop interaction. In 2017 IEEE Int. Conf. on Robotics and Automation (ICRA), Singapore, 29 May–3 June, pp. 4449–4456. IEEE (10.1109/ICRA.2017.7989515)

[14] Calvo Garzón P, Keijzer F 2011 Plants: adaptive behavior, root-brains, and minimal cognition. Adapt. Behav. 19, 155–171. (10.1177/1059712311409446)

[15] Wahby M, Hofstadler DN, Heinrich MK, Zahadat P, Hamann H 2016 An evolutionary robotics approach to the control of plant growth and motion: modeling plants and crossing the reality gap. In 2016 IEEE 10th Int. Conf. on Self-Adaptive and Self-Organizing Systems (SASO), Augsburg, Germany, 12–16 September, pp. 21–30. IEEE (10.1109/SASO.2016.8)

[16] Hofstadler DN, Wahby M, Heinrich MK, Hamann H, Zahadat P, Ayres P, Schmickl T 2017 Evolved control of natural plants: crossing the reality gap for user-defined steering of growth and motion. ACM Trans. Auton. Adapt. Syst. (TAAS) 12, 1–24. (10.1145/3124643)

[17] Wahby M, Heinrich MK, Hofstadler DN, Zahadat P, Risi S, Ayres P, Schmickl T, Hamann H 2018 A robot to shape your natural plant: the machine learning approach to model and control bio-hybrid systems. In Proc. of the Genetic and Evolutionary Computation Conf., GECCO ’18, Kyoto, Japan, 15–19 July, pp. 165–172. New York, NY: ACM (10.1145/3205455.3205516)

[18] Hamann H *et al.* 2015 *flora robotica*—mixed societies of symbiotic robot-plant bio-hybrids. In Proc. of IEEE Symposium on Computational Intelligence (IEEE SSCI 2015), Cape Town, South Africa, 7–10 December, pp. 1102–1109. IEEE (10.1109/SSCI.2015.158)

[19] Hamann H *et al.* 2017 *flora robotica*—an architectural system combining living natural plants and distributed robots. (http://arxiv.org/abs/1709.04291)

[20] Al-Beeshi B, Al-Mesbah B, Al-Dosari S, El-Abd M 2015 iPlant: the greenhouse robot. In IEEE 28th Canadian Conf. on Electrical and Computer Engineering (CCECE), Halifax, Canada, 3–6 May, pp. 1489–1494. IEEE (10.1109/CCECE.2015.7129501)

[21] Zabel P, Bamsey M, Schubert D, Tajmar M 2016 Review and analysis of over 40 years of space plant growth systems. Life Sci. Space Res. 10, 1–16. (10.1016/j.lssr.2016.06.004) 27662782

[22] Agostini A, Alenyá G, Fischbach A, Scharr H, Wörgötter F, Torras C 2017 A cognitive architecture for automatic gardening. Comput. Electron. Agri. 138, 69–79. (10.1016/j.compag.2017.04.015)

[23] Burris JN, Lenaghan SC, Stewart CN 2018 Climbing plants: attachment adaptations and bioinspired innovations. Plant Cell Rep. 37, 565–574. (10.1007/s00299-017-2240-y) 29188422

[24] Barthlott W, Mail M, Bhushan B, Koch K 2017 Plant surfaces: structures and functions for biomimetic innovations. Nano-Micro Lett. 9, 23 (10.1007/s40820-016-0125-1) PMC6223843 30464998

[25] Vidoni R, Mimmo T, Pandolfi C 2015 Tendril-based climbing plants to model, simulate and create bio-inspired robotic systems. J. Bionic Eng. 12, 250–262. (10.1016/s1672-6529(14)60117-7)

[26] Cortese L, Milanovic S, Vidoni R 2017 A FEM-experimental approach for the development of a conceptual linear actuator based on tendril’s free coiling. Appl. Bionics Biomech. 2017, 1–12. (10.1155/2017/6450949) PMC5547720 28811739

[27] Fiorello I, Tricinci O, Mishra AK, Tramacere F, Filippeschi C, Mazzolai B 2018 Artificial system inspired by climbing mechanism of *galium aparine* fabricated via 3D laser lithography. In Conf. on Biomimetic and Biohybrid Systems, Paris, France, 17–20 July, pp. 168–178. Cham, Switzerland: Springer.

[28] Park SJ *et al.* 2016 Phototactic guidance of a tissue-engineered soft-robotic ray. Science 353, 158–162. (10.1126/science.aaf4292) 27387948 PMC5526330

[29] Tina S, Danninger E, Harder D, Speck T, Kraft O, Schwaiger R 2010 Quantifying the attachment strength of climbing plants: a new approach. Acta Biomater. 6, 1497–1504. (10.1016/j.actbio.2009.10.003) 19818882

[30] Oxman N, Laucks J, Kayser M, Duro-Royo J, Gonzales-Uribe C 2014 Silk pavilion: a case study in fiber-based digital fabrication. In FABRICATE Conf. Proc, Zurich, Switzerland, pp. 248–255. London, UK: UCL Press.

[31] Elsayed S, Boukis N, Sauer J, Patzelt D, Kerner M, Hindersin S 2015 Algal cultivation and hydrothermal gasification: biomass and energy production. ET. Energiewirtschaftliche Tagesfragen 65, 2–4.

[32] Nagy D, Locke J, Benjamin D 2015 Computational brick stacking for constructing free-form structures. In *Modelling Behaviour*, pp. 203–212. Berlin, Germany: Springer (10.1007/978-3-319-24208-8_17)

[33] Torgal FP, Labrincha JA, Vittoria Diamanti M, Yu C-P, Lee H-K 2015 Biotechnologies and biomimetics for civil engineering. Berlin, Germany: Springer.

[34] Ludwig F, Schwertfeger H, Storz O 2012 Living systems: designing growth in Baubotanik. Archit. Des. 82, 82–87. (10.1002/ad.1383)

[35] Schmitz V 2016 Möbel aus dem Garten: Einzigartiges Naturdesign bei Full Grown aus England. AIT 2016, 142–143.

[36] Gale B 2011 The potential of living willow structures in the landscape. Master’s thesis, State University of New York, College of Environmental Science and Forestry.

[37] Reames R 2005 Arborsculpture: solutions for a small planet. Williams, OR: Arborsmith Studios.

[38] Kirsch K 1997 Naturbauten aus lebenden Gehölzen. Xanten, Germany: OLV, Organischer Landbau-Verlag Lau.

[39] Wiechula A 1928 Verfahren zum Vereinigen von Baumteilen; A method for combining parts of trees, May 18 1928. DE Patent 459,996.

[40] Paul G 1998 Origin and evolution of common bean: past events and recent trends. HortScience 33, 1124–1130.

[41] Gianoli E 2015 The behavioural ecology of climbing plants. AoB Plants 7, plv013 (10.1093/aobpla/plv013) 25678588 PMC4363473

[42] Migliaccio F, Tassone P, Fortunati A 2013 Circumnutation as an autonomous root movement in plants. Am. J. Botany 100, 4–13. (10.3732/ajb.1200314) 23243099

[43] Mugnai S, Azzarello E, Masi E, Pandolfi C, Mancuso S 2015 Nutation in plants. In *Rhythms in plants: dynamic responses in a dynamic environment* (eds Mancuso S, Shabala S), ch. 2, pp. 19–34. Cham, Switzerland: Springer International Publishing (10.1007/978-3-319-20517-5_2)

[44] Schrank AR 1950 Plant tropisms. Ann. Rev. Plant Physiol. 1, 59–74. (10.1146/annurev.pp.01.060150.000423)

[45] Bastien R, Meroz Y 2016 The kinematics of plant nutation reveals a simple relation between curvature and the orientation of differential growth. PLoS Comput. Biol. 12, e1005238 (10.1371/journal.pcbi.1005238) 27923062 PMC5140061

[46] Baldwin KL, Strohm AK, Masson PH 2013 Gravity sensing and signal transduction in vascular plant primary roots. Am. J. Botany 100, 126–142. (10.3732/ajb.1200318) 23048015

[47] Vandenbrink JP, Kiss JZ 2016 Space, the final frontier: a critical review of recent experiments performed in microgravity. Plant Sci. 243, 115–119. (10.1016/j.plantsci.2015.11.004) 26795156 PMC5739877

[48] Liscum E, Askinosie SK, Leuchtman DL, Morrow J, Willenburg KT, Coats DR 2014 Phototropism: growing towards an understanding of plant movement. Plant Cell 26, 38–55. (10.1105/tpc.113.119727) 24481074 PMC3963583

[49] Hughes J 2013 Phytochrome cytoplasmic signaling. Plant Biol. 64, 377–402. (10.1146/annurev-arplant-050312-120045) 23506333

[50] Galvão VC, Fankhauser C 2015 Sensing the light environment in plants: photoreceptors and early signaling steps. Curr. Opin Neurobiol. 34, 46–53. (10.1016/j.conb.2015.01.013) 25638281

[51] Ouzounis T, Rosenqvist E, Ottosen CO 2015 Spectral effects of artificial light on plant physiology and secondary metabolism: a review. HortScience 50, 1128–1135.

[52] Hogewoning SW, Wientjes E, Douwstra P, Trouwborst G, van Ieperen W, Croce R, Harbinson J 2012 Photosynthetic quantum yield dynamics: from photosystems to leaves. Plant Cell 24, 1921–1935. (10.1105/tpc.112.097972) 22623496 PMC3442578

[53] Christie JM, Murphy AS 2013 Shoot phototropism in higher plants: new light through old concepts. Am. J. Botany 100, 35–46. (10.3732/ajb.1200340) 23048016

[54] Briggs WR 2014 Phototropism: some history, some puzzles, and a look ahead. Plant Physiol. 164, 13–23. (10.1104/pp.113.230573) 24399823 PMC3875795

[55] Pierik R, De Wit M 2014 Shade avoidance: phytochrome signalling and other aboveground neighbour detection cues. J. Exp. Bot. 65, 2815–2824. (10.1093/jxb/ert389) 24323503

[56] Fraser DP, Hayes S, Franklin KA 2016 Photoreceptor crosstalk in shade avoidance. Curr. Opin. Plant Biol. 33, 1–7. (10.1016/j.pbi.2016.03.008) 27060719

[57] Wang Y, Folta KM 2013 Contributions of green light to plant growth and development. Am. J. Botany 100, 70–78. (10.3732/ajb.1200354) 23281393

[58] Müller D, Waldie T, Miyawaki K, To JPC, Melnyk CW, Kieber JJ, Kakimoto T, Leyser O 2015 Cytokinin is required for escape but not release from auxin mediated apical dominance. Plant J. 82, 874–886. (10.1111/tpj.12862) 25904120 PMC4691322

[59] Borghi L, Liu GW, Emonet A, Kretzschmar T, Martinoia E 2016 The importance of strigolactone transport regulation for symbiotic signaling and shoot branching. Planta 243, 1351–1360. (10.1007/s00425-016-2503-9) 27040840 PMC4875938

[60] Ali MM, Moon KS 2007 Structural developments in tall buildings: current trends and future prospects. Archit. Sci. Rev. 50, 205–223. (10.3763/asre.2007.5027)

[61] Moon KS 2011 Diagrid structures for complex-shaped tall buildings. Procedia Eng. 14, 1343–1350. (10.1016/j.proeng.2011.07.169)

[62] Milana G, Gkoumas K, Bontempi F 2014 Sustainability concepts in the design of high-rise buildings: the case of diagrid systems. In Proc. of the 3rd Int. Workshop on Design in Civil and Environmental Engineering, Kongens Lyngby, Denmark, 22–23 August, pp. 170–179. Kongens Lyngby, Denmark: Technical University of Denmark (10.5072/dtu:2913)

[63] McCree KJ 1971 The action spectrum, absorptance and quantum yield of photosynthesis in crop plants. Agri. Meteorol. 9, 191–216. (10.1016/0002-1571(71)90022-7)

[64] Wahby M, Heinrich MK, Hofstadler DN, Neufeld E, Kuksin I, Zahadat P, Schmickl T, Ayres P, Hamann H 2018 Data from: Autonomously shaping natural climbing plants: a bio-hybrid approach Zenodo. (10.5281/zenodo.1172160). PMC6227980 30473806

